# Metastatic Insulinoma in a Patient with Type 2 Diabetes Mellitus: Case Report and Review of the Literature

**DOI:** 10.1155/2011/124078

**Published:** 2011-02-10

**Authors:** Noormuhammad Oosman Abbasakoor, Marie Louise Healy, Donal O'Shea, Donal Maguire, Cian Muldoon, Kieran Sheahan, Dermot O'Toole

**Affiliations:** ^1^Department of Endocrinology, St. James' Hospital, Dublin 8, Ireland; ^2^Department of Endocrinology, St. Vincent's University Hospital, Dublin 4, Ireland; ^3^Department of Surgery, St. Vincent's University Hospital, Dublin 4, Ireland; ^4^Department of Pathology, St. James' Hospital, Dublin 8, Ireland; ^5^Department of Pathology, St. Vincent's University Hospital, Dublin 4, Ireland; ^6^Department of Gastroenterology, St. James' Hospital, Dublin 8, Ireland

## Abstract

Pancreatic neuroendocrine tumors (NETs) are extremely rare, and although insulinomas are the commonest, less than 10% of insulinomas are malignant. Most patients with insulinomas present with neuroglycopenic symptoms and weight gain attributable to insulin excess. Here, we report a case where a 67-year-old lady with a background history of type 2 diabetes mellitus and breakthrough hyperinsulinism who presented with coma. The biochemical profile revealed features typical of insulinoma, and CT and endosonography confirmed a pancreatic tumor with large volume right-sided liver metastases (biopsy confirming a neuroendocrine tumor). The patient underwent successful one-step RO surgical resection, distal pancreatectomy, splenectomy, and right hepatectomy, and 9 months postoperatively, she remains free of recurrent disease. She remains a diabetic.

## 1. Introduction

Insulinoma is a rare tumour, derived from the beta cells of the pancreas. The incidence in the general population is 4 cases per million a year [1]. 80% to 90% of insulinomas are benign, and less than 10% are malignant. Insulinoma is the most common form of pancreatic NET accounting for 60% of cases [2] and is most commonly seen between the age of 40 to 60 years, in females (60%) [1, 3]. They usually present with symptoms of hyperinsulinism resulting in neuroglycopenia such as headache, diplopia, blurred vision, confusion, abnormal behaviour, lethargy, and amnesia. Rarely, hypoglycemia may result in seizures and coma. Other features of hyperinsulinism result from the autonomic nervous system including sweating, weakness, hunger, tremor, nausea, feelings of warmth, anxiety, and palpitations [4]. The symptoms resolve with dextrose infusion and with food intake. The association of insulinoma in a malignant form and diabetes mellitus is a rarely reported phenomenon as presented in the following report. The report also illustrates how medical management rapidly failed and also how an aggressive surgical strategy proved to be clinically and oncologically feasible.

## 2. Case Report

A 67-year-old lady was admitted via the emergency department after being found unresponsive at home by her husband early in the morning. On presentation, her GCS was 9 with normal vital signs. Her glucose level was 8 mmol/L, and the blood gases were normal. On observation an hour later, her dextrose test was rechecked and fell to 1.5 mmol/L with blood glucose of 0.9 mmol/L. She was given intravenous dextrose and her condition improved.

Of note, her past medical history included a two-year history of type 2 diabetes mellitus, recently diagnosed depression, and osteoporosis. Her oral hypoglycemic agents had been recently withdrawn as her blood sugar levels were well controlled on dietary changes she had made. Her family reports that she was increasingly anxious over the last few months with occasional confused periods. She also had a 4 kg weight loss over the last 12 months (10% body weight). Her daily medications include citalopram, bromazepam, and weekly bisphosphonate. There was no family history of diabetes mellitus, hypertension, or hyperparathyroidism. The patient denied taking any excess medication or oral hypoglycemic agents.

On examination, the patient was alert but appeared very anxious; her GCS returned rapidly to normal on intravenous dextrose. Cardiovascular, gastrointestinal, and neurological examinations were normal as well as her other laboratory investigations.

The patient underwent a 72-hour fast as part of the investigation but was interrupted at 2 hours due to hypoglycemic symptoms (sweating, feeling drowsy and unwell). During this episode, her blood glucose level was confirmed to be low (1.1 mmol/L), C peptide was elevated at 8.2 and insulin levels at 91. Her sulfonylurea screen was negative and her other pancreatic endocrine hormones were sent and came back as within normal limits.

**Figure 1:**
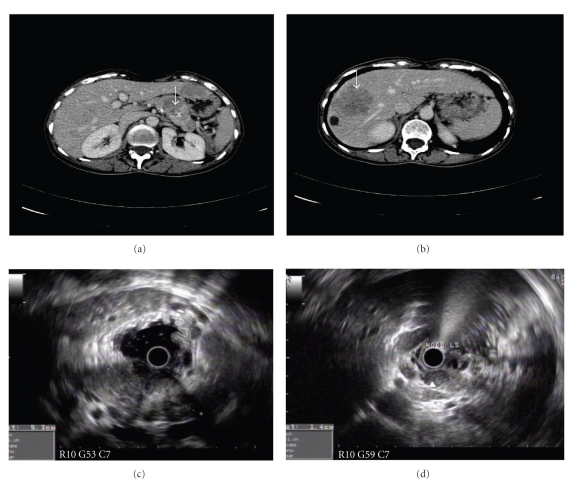
MD-CT scan demonstrating a hypodense lesion (arrow) with spots of peripheral calcifications in the body/tail of pancreas as wells as a large hypodense liver metastasis in the right lobe of liver, arrow (b). (c, d) show at endoscopic ultrasound the presence of the heterogeneous hypoechoic mass involving the body and tail of pancreas with numerous hypoechoic well defined peri-pancreatic lymph nodes (d).

**Figure 2:**
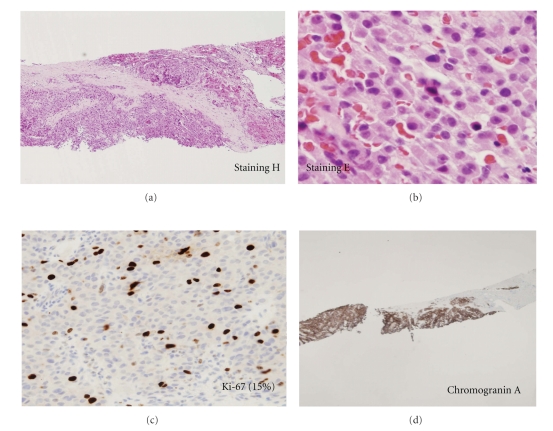
US-guided liver biopsy staining H & E (a) demonstrating a rather uniform population of sheets of small cells arranged in manner (×40 in (b)). Immunohistochemistry with a Ki-67 marker (Mib-1) (c) estimated here to be 15% and positive immunostaining with chromogranin A (d).

CT scan of abdomen revealed a 2.6 cm by 2.0 cm hypodense mass containing peripheral calcifications in the body of the pancreas (Figure 1). Multiple low attenuation liver lesions, with the largest one measuring 5 cm, were seen in segment 8 of the liver. An endoscopic ultrasound (EUS) also found a 5- to 6-cm oval-shaped hypoechoic lesion (Figure 1) with irregular margins involving the left body and junction body/tail of the pancreas clearing invading the splenic artery with several hypoechoic large local lymph nodes. EUS-guided biopsies of the pancreatic mass and nodes were performed. A somatostatin receptor scintigraphy with octreotide showed intense uptake in the pancreas and the liver and no distant lesions. Due to the aggressive nature of the clinical presentation with anorexia and weight loss, an ultrasound-guided liver biopsy of hepatic metastases was also performed. Histopathology from all sites confirmed neuroendocrine differentiation with diffuse chromogranin A and synaptophysin staining; the Ki-67 proliferation index varied form <2% in the pancreas to 10 to 15% in liver metastases (Figure 2).

The initial management consisted of intravenous dextrose infusions for multiple episodes of hypoglycemia with no particular diurnal variations, and these episodes were not relieved postprandially. Oral diazoxide was rapidly commenced without any appreciable effect on hypoglycemic episodes, and the patient was thereafter commenced on subcutaneous octreotide (100 mcg three times a day increasing to 600 mcg/day); the latter improved marginally the glucose levels, but she developed peripheral oedema and abdominal cramps. Following a multidisciplinary discussion, it was decided to attempt surgery using either a one-stage or two-stage strategy. At laparotomy, she underwent a distal pancreatectomy, splenectomy, and right hepatectomy. Wedge excision of segment 2 was also performed at the time of the procedure. The postoperative period was marked by difficult to control hyperglycemia, but the patient made a rapid recovery and was fit to discharge home after 14 days. All symptoms attributable to hypoglycemia completely disappeared with the patient experiencing a normal psychological state—no episodes of anxiety or unexplained confusion or stress as described prior to presentation. She is in good health with normal follow-up imaging, C-peptide levels measurement. She remains diabetic nine months postoperatively, and the diabetes is diet controlled. 

## 3. Discussion

Insulinomas are insulin-secreting tumours from the beta cells of the pancreas. 80% of patients diagnosed with insulinomas have single, usually small (60%–75% are less than (<) 1.5 cm), benign (TNM Stage 1 or stage IIa) tumours [5]. Only 10% have malignant tumours and the remaining 10% have multiple benign lesions [6, 7]. Approximately 40% of the tumours are <1 cm in diameter and can be as small as 2 mm and difficult to detect [7]. Malignant tumours tend to be larger and >3 cm in diameter as was the case here [7]. The clinical and biochemical pattern as depicted by this case report fit with the classical patterns described in the literature [8].

Additionally, the imaging features here did not pose any particular difficulties with typical features on CT and endosonography. Somatostatin receptor scintigraphy is often negative in the detection of small benign tumours (40 to 50%) [8]. In malignant disease, positive scintigraphy is described due to a different relative distribution of somatostatin receptor subtypes and a higher rate of scan-positivity with this technique can be expected [9–11], and this can be useful in planning management.

Insulinomas occurring in diabetics is very rare, and there are very few published reports in the literature. Between 1927 and 1992, there was only one case of insulinoma occurring in a diabetic patient, out of the 313 confirmed cases of insulinomas in Mayo Clinic series [12, 13]. In Japan, a review of 443 cases of insulinomas between 1976 and 1990 revealed only one patient who had coexisting diabetes [12, 14]. In one institution in Taiwan, out of 23 cases of insulinomas seen between July 1984 and March 2006, only one case of coexisting diabetes was recorded [12].

The management of insulinoma depends on tumour localisation, the malignant potential, and presence of metastasis. Medical treatment for insulinomas is indicated for difficult to control hypoglycemia symptoms usually while awaiting more definite therapeutic strategies. Diazoxide (50–300 mg/day which can be increased up to 600 mg/Day), which inhibits insulin secretion from the pancreatic beta cells and stimulates gluconeogenesis, has been used successfully to some extent for symptom control while awaiting more definitive management [15]. The main side effects include palpitations, oedema, and hirsutism and they occurred in 47% of patients on diazoxide [16]. A thiazide diuretic is usually used both to reduce oedema and provide a hyperglycemic effect [16]. Diazoxide is particularly helpful and used in patients with tumour nonlocalisation, failed surgery, metastatic disease, and patients unfit for surgery [16]. The beneficial effects are often however short-lived. Somatostatin analogues can also be employed due to their inhibitory effects on glucagon and growth hormone secretion and lowers endogenous insulin production. The main mechanism of action of somatostatin analogues in insulinoma is the inhibition of insulin secretion by beta cells of the pancreas. This is achieved by binding to somatostatin (SRIF) receptors mainly sst2 and sst5 which are expressed by beta cells [11]. Traditional systemic cytotoxic combinations have been disappointing in managing patients with metastatic disease [17]. Recently, case reports on both the control of hypoglycemia and even objective response have been reported in patients with metastatic disease using an mTOR (mammalian target of rapamycin) inhibitor—everolimus [18, 19]. More data are required to substantiate these interesting findings.

Surgery is the recommended treatment in most cases of insulinomas with limited resection recommended for single small lesions [8]. Management of malignant insulinomas should include an oncological resection where possible and attempts at RO (complete resection with no microscopic residual tumour) resection including resection of liver metastases [20]. We decided to embark on either a one- or two-stage resection in this case; planned oncological resection of the diseased pancreas and nodes and if the liver metastases were not resectable at that time, portal embolisation to the right followed by right hepatectomy at a later date was planned as described [21]. Fortunately, a one-stage strategy was possible here with a rapid recovery; to the extent that postoperative hyperglycemia was difficult to control. The median disease-free survival after curative resection is 5 years, but recurrence occurs in more than 60% at a median interval of 2.5–3 years. Median survival with recurrent tumours is less than 2 years [22]. Palliative resection may prolong median survival [8]. If an RO resection is not deemed possible, then debulking procedures can be considered to aid symptomatic control. Other strategies include: radiofrequency ablation [23], and transarterial chemoembolisation [23] or peptide receptor radionuclide therapy [24]. Based on all previous literature, the better survival rates were associated with resection of primary tumour and intention to treat metastatic disease [25].

## 4. Conclusion

Metastatic insulinoma in a diabetic patient is an extremely rare disease. Hypoglycemia in a diabetic patient following withdrawal of oral hypoglycemic agents with associated improvement in diabetes status should be investigated for insulinoma. Surgical resection should always be considered where possible for insulinomas whether malignant or not. 

##  Conflict of Interests

The authors declare that there is no conflict of interest that could be perceived as prejudicing the impartiality of the research reported. 

##  Disclosure

The authors hereby confirm that neither the paper nor any part of it, except for abstracts of less than 400 words, has been published or is being considered for publication elsewhere. By signing this letter, each of them acknowledges that he or she participated sufficiently in the work to take public responsibility for its consent.
